# On-Line Mixture Quantification to Track Temporal Change of Composition Using FAIMS

**DOI:** 10.3390/s19245442

**Published:** 2019-12-10

**Authors:** Yasufumi Yokoshiki, Takamichi Nakamoto

**Affiliations:** 1 Department of Information and Communications Engineering, School of Engineering, Tokyo Institute of Technology, Yokohama-shi, Kanagawa 226-8503, Japan; yokoshiki.y.aa@m.titech.ac.jp; 2 Laboratory for Future Interdisciplinary Research of Science and Technology, Institute of Innovative Research, Tokyo Institute of Technology, Yokohama-shi, Kanagawa 226-8503, Japan

**Keywords:** quantification of gas mixtures, faims, odor, e-nose

## Abstract

This paper reports on-line mixture quantification with FAIMS. Ternary gas mixtures composed of acetone, ethanol, and diethyl ether were used for quantification. We succeeded in an on-line quantification of ppm-level concentration and even sub-ppm-level gases using the gradient descent method. It took 10 minutes to quantify the ternary mixture. However, it was too long, because we aim to track the temporal change of each component concentration in the mixture. Then, an algorithm based on feedback control was introduced to reduce the quantification time. The feedback method successfully tracked concentrations in three cases. The simulation result shows that the proposed method can reduce the quantification time.

## 1. Introduction

Nowadays, gas chromatography-mass spectrometry (GC-MS) is a standard method of gas analysis for chemical quantification [1,2,3]. Direct-MS is used to sample analytes directly because of its fast speed and high sensitivity [4]. Selected ion flow tube mass spectrometry (SIFT-MS) is a kind of direct-MS that is used for real-time detection and quantification because it does not require trapping, preconcentration, pretreatments, or separation [5,6]. Proton transfer reaction mass spectrometry (PTR-MS), which is also another type of direct-MS, has high sensitivity [7]. Proton transfer reaction time of flight mass spectrometry (PTR-TOF-MS) derived from PTR-MS has a sub-ppt-level detection limit [8].

Ion mobility spectrometry (IMS) also has a long history of research [9,10]. The mechanism is entirely different from MS [11], although the structure of IMS is similar to MS. IMS uses the information of mobility as a function of the collision cross-section, which is a measure of the ion size. In comparison to IMS, MS measures the weight per ionic valence of ionized analyte in a vacuum as a spectrum. Field asymmetric ion mobility spectrometry (FAIMS) is a type of IMS [12,13]. IMS detects ions with the time difference, whereas FAIMS measures ions by sweeping voltage between electrodes. The field is called the dispersion field (DF), whereas the bias voltage is called the compensation voltage (CV). DF is an amplitude of an asymmetric square wave. FAIMS has advantages over GC-MS because of low cost, no vacuum required, and fast response. Moreover, FAIMS may obtain information related to the collision cross-section, which may have other feature information different from the information of the mass-to-charge ratio in MS. Direct-MS has higher sensitivity and a shorter detection time than FAIMS [14]. However, FAIMS may have the advantage of the cost and ease of maintenance due to the simple structure.

FAIMS has been applied to many situations. Some papers reported real-time detection with FAIMS for diseases. Sinha et al. performed real-time infection detection for potatoes and onions [15], Osmo et al. certificated that FAIMS can separate the ions from noises in real-time measurement based on the concept of Shannon entropy [16]. However, the feasibility of real-time mixture quantification with FAIMS has not been confirmed. The quantification method using off-line measurement data was reported in the previous paper [17]. In this study, the on-line measurement system was developed as an extension from the previous system, and trinary gas mixtures were utilized to test the quantification performance.

It can be applied to the real-time sensing such as the odor tracking of temporal change of each component concentration in the mixture. An odor replication system reported previously tracked the temporal change of concentrations of some odorants [18]. There are some researches related to the temporal change of gas mixture concentrations. Erin et al. used planar laser-induced fluorescence for the quantification of acetone in a broad area [19]. Ilitani et al. developed a gas-imaging system that can obtain the concentration-distribution of acetaldehyde in breath and released from a palm skin [20]. This application will be useful for odor source tracking. Junji et al. reported an odor tracking robot using a biosensor obtained from male silk moth [21]. Tanthip et al. demonstrated a humanoid robot with wearable sensors based on Polymer/Carbon nanotubes for odor tracking [22]. We aim to establish the fundamental method of tracking a temporal change of each concentration in the mixture using FAIMS in the present study.

## 2. Materials and Methods

**Figure 1:**
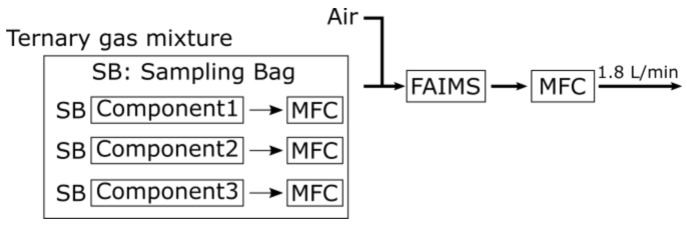
The block diagram of the experimental system.

Figure 1 shows a brief explanation of the experimental system where the mixture composition is determined by the flowrates of mass flow controllers (MFCs). The sampling bag was directly connected to MFC, and the vapor inside the bag flows owing to the negative pressure caused by the air pump. The mixture composition is determined by the ratio of each component flowrate.

Some improvements to realize the on-line system extensions will be explained below. There is a difference of protocol between this research and the previous one. Table 1 shows the protocol of quantification in previous research, whereas the protocol of on-line quantification using FAIMS is explained in Table 2. The measurement protocol is shown in Table 3. Details were written in previous work [17].

Some definitions are used in this section. Measurement points are defined as all of the points measured with FAIMS for each on-line measurement. The update point is defined as a specific point that moves to the direction of the steepest gradient and approaches the solution little by little. A collected point is defined as the points needed for the calculation of gradients. The error hypersurface is defined as the three-dimensional hypersurface obtained from indices *E* explained in Equation (2) as a function of concentrations. Lattice points comprise a regularly spaced array of concentration points.

### 2.1. Data Preprocessing

A simple noise elimination method was utilized because data measured with FAIMS have some noise. The noise without signal was eliminated by using a threshold value. The threshold value was empirically set at 0.08, whereas the data range was between 0 and 10.

### 2.2. Quantization for Quantification

**Figure 2:**
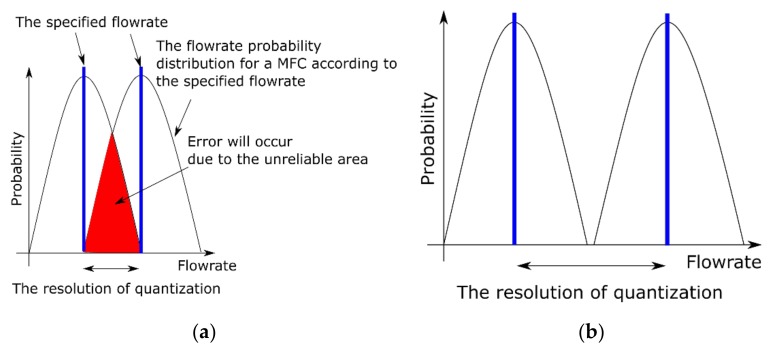
The difference between a specified flowrate and an actual flowrate. The actual flowrates fluctuate within a certain level. If a resolution of quantization is inappropriate, the distribution of the different flowrates overlap, and this will cause an error. The distributions next to each other should not overlap. (**a**) Inappropriate resolution. (**b**) Appropriate resolution.

**Figure 3:**
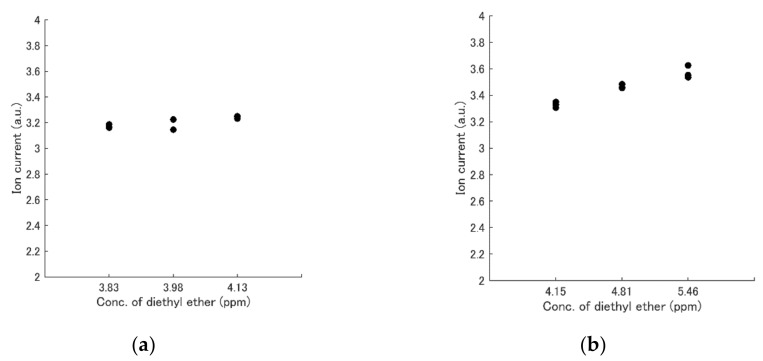
Repeatability of FAIMS ion current. (**a**) 0.5% resolution. (**b**) 2% resolution. The concentration of diethyl ether was changed, whereas the acetone and ethanol concentrations were 3.0 ppm and 3.0 ppm, respectively. The highest value along the line DF = 40% was plotted in each case.

Every MFC has a certain fluctuation level of its flowrate because a MFC controls the flowrate using feedback. The fluctuation of flowrate deteriorates the repeatability of data (Figure 2). Therefore, the flowrate was quantized every 2% of its full scale so that a small concentration difference could be clearly distinguished, as is shown in Figure 3. It is essential to keep the resolution at an appropriate level, since we need to approximate the derivative of the ion current with respect to concentration when we use the gradient descent method. Each flowrate can be converted to concentration using the equation:

(1) $$C=\frac{\alpha V_{\max}C_{S}}{V_{total}}\;\left(\mathrm{ppm}\right),$$

where α (= 0.02) is the relative resolution, $V_{\max}$ is the maximum flowrate of MFC (L/min), $C_{S}$ is the concentration of a gas in a sampling bag, and $V_{total}$ (= 1.8 L/min) is the total flowrate at the FAIMS device. Figure 3 shows that the 2% resolution was appropriate, whereas one of 0.5% was insufficient.

### 2.3. Local Minimum Detection Algorithm

**Figure 4:**
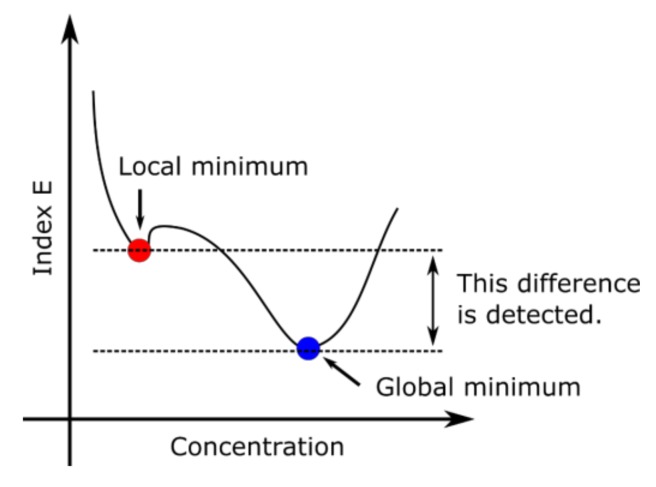
Explanation of local minimum and global minimum. Local minimum and global minimum can be distinguished using the difference of Index *E*.

The difference value between the target data and measured data was utilized for local minimum detection (see Figure 4). The well-known problem with gradient descent is a local minimum. The correct solution is called a global minimum. However, information about the gradient sometimes leads to the local minimum. The index *E* can be calculated as

(2) $$E=\overset{k}{\underset{i=1}{\sum }}\overset{l}{\underset{j=1}{\sum }}\left|IC_{\left(i,j\right)}-IC_{target\left(i,j\right)}\right|$$

to distinguish between local and global minima, where k is the number of DFs, l is the number of CVs, IC is the ion current matrix obtained from each measured data, and $IC_{target}$ is the ion current matrix obtained from the data at target concentrations. If the position of the local minimum is far from the global minimum, the value of E tends to be larger than the noise level. If the position of the local minimum is near the target point, the E approaches zero. Therefore, if the E is still large after stagnation is found, the exploration continues from a different point. If the E becomes below the threshold, the exploration is stopped in order to reduce the time required for quantification. The threshold value was empirically selected because it depends on the component concentrations of the ternary gas mixture.

### 2.4. Data Collected Point

**Figure 5:**
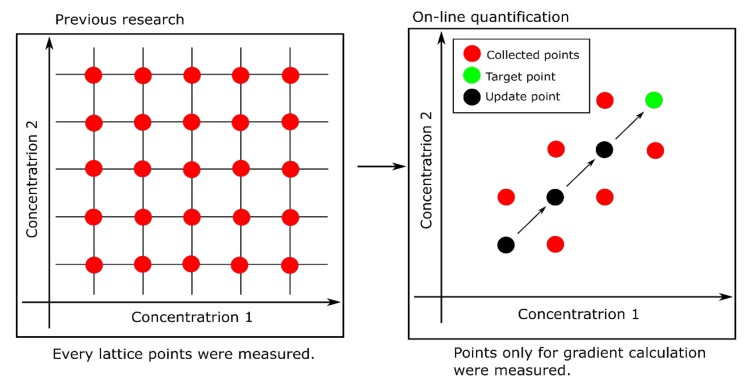
Explanation of decrease measurement points. On-line quantification measured the points needed for only gradient calculation, whereas every lattice point was measured in previous research.

**Figure 6:**
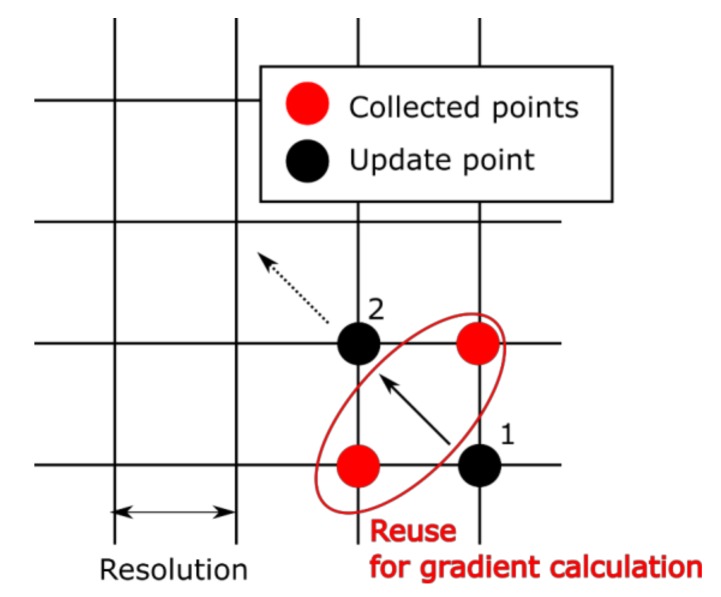
Explanation of the reuse of collected points. When there are collected points near the update points, those were reused for gradient calculation.

This system was improved to obtain only several data points required for gradients calculation, whereas all possible combinations of component concentrations were measured in the previous work in advance [17] (see Figure 5). However, it took a long time due to increasing measurement points (it took almost three hours). If only the points required for the calculation of the gradients can be considered, the time decreases drastically. Moreover, if measured points are reused for the calculation, the time will be further reduced. However, an excessive decrease in the number of measured points deteriorates the accuracy of gradient estimation. Neighbors of the update points were reused to satisfy the trade-off relation between the accuracy and the quantification time (see Figure 6).

### 2.5. Mechanism of Concentration-Change Track Using Feedback

Tracking the temporal change of each component concentration using the feedback was tested with quartz crystal microbalance (QCM) sensors previously [23]. The mechanism will be explained below. When the sensor responses are approximated with the first-order delay, the dynamics is expressed using

(3) $$s_{k+1}=Fs_{k}+Gu_{k},$$

where $s_{k}$ is the sensor response vector at the time *k*Δt, $u_{k}$ is the component concentration change vector at the time *k*Δt, and Δt is the period of the sensor response. The $s_{k}={\left[s_{1}\left(k\right),\;\ldots ,\;s_{i}\left(k\right),\ldots ,s_{n}\left(k\right)\right]}^{T}$, $u_{k}={\left[u_{1}\left(k\right),\;\ldots ,u_{j}\left(k\right),\;\ldots ,\;u_{m}\left(k\right)\right]}^{T}={\left[c_{1}\left(k\right)-c_{1}\left(k-1\right),\;\ldots ,c_{j}\left(k\right)-c_{j}\left(k-1\right),\;\ldots ,\;c_{m}\left(k\right)-c_{m}\left(k-1\right)\right]}^{T}$, where $s_{i}\left(k\right)$ is the $i_{th}$ sensor response at the time *k*, $u_{j}\left(k\right)$ is the $j_{th}$ the component concentration change at the time *k*, and $c_{j}\left(k\right)$ is $j_{th}$ the component concentration at the time *k*. Equation (3) is expressed as the state-space equation for a discrete time system using the response vector $s_{k}$ and concentration-change vector $u_{k}$ at the time k. When F, and ***G*** are represented by

(4) $$F=\left[\begin{array}{ccc} f_{1} & & 0\\ & \ddots & \\ 0 & & f_{n} \end{array}\right],\;G=\left[\begin{array}{ccc} g_{11} & \cdots & g_{1m}\\ \vdots & \ddots & \vdots \\ g_{n1} & \cdots & g_{nm} \end{array}\right],$$

Equation (3) can be written in the next equation for the $i_{th}$ sensor:

(5) $$s_{i}\left(k+1\right)=\left[f_{i}\;g_{i1}\;\cdots g_{im}\right]\left[\begin{array}{c} s_{i}\left(k\right)\\ \begin{array}{c} c_{1}\left(k\right)-c_{1}\left(k-1\right)\\ \vdots \end{array}\\ c_{m}\left(k\right)-c_{m}\left(k-1\right) \end{array}\right].$$

Therefore, the equation below is derived using data from *k =* 1 to *k = N*:

(6) $$\left[\begin{array}{c} s_{i}\left(2\right)\\ \vdots \\ s_{i}\left(N\right) \end{array}\right]=\left[\begin{array}{cccc} s_{i}\left(1\right) & c_{1}\left(1\right)-c_{1}\left(0\right) & \cdots & c_{m}\left(1\right)-c_{m}\left(0\right)\\ \vdots & \vdots & & \vdots \\ s_{i}\left(N-1\right) & c_{1}\left(N-1\right)-c_{1}\left(N-2\right) & \cdots & c_{m}\left(N-1\right)-c_{m}\left(N-2\right) \end{array}\right]\left[\begin{array}{c} f_{i}\\ g_{i1}\\ \vdots \\ g_{im} \end{array}\right]=\;D_{i}\left[\begin{array}{c} f_{i}\\ g_{i1}\\ \vdots \\ g_{im} \end{array}\right].$$

The parameter vector can be obtained from the least-squares solution expressed by the following equation:

(7) $$\left[\begin{array}{c} f_{i}\\ g_{i1}\\ \vdots \\ g_{im} \end{array}\right]={\left(D_{i}^{T}D_{i}\right)}^{-1}D_{i}^{T}\left[\begin{array}{c} s_{i}\left(2\right)\\ \vdots \\ s_{i}\left(N\right) \end{array}\right].$$

Thus, all parameters in Equation (3) can be obtained from Equation (7).

### 2.6. Optimal Feedback Control

The feedback is controlled by the index value *J*:

(8) $$J=\overset{p-1}{\underset{k=0}{\sum }}\{{\left(s_{k+1}-s_{target}\right)}^{T}Q\left(s_{k+1}-s_{target}\right)+u_{k}^{T}Ru_{k}\},$$

where $s_{k}$ is the sensor response vector to the blended at time *k*Δt, $s_{target}$ is the sensor response to the target gas mixture, and *p* is the number of concentration-change steps during the feedback. The first term in Equation (8) is the summation of squares of the differences between the sensor response to the gas mixture at time *k*Δt and the sensor response to the target gas mixture. The difference is weighted by a diagonal matrix ***Q***. The second term is the summation of squares of the concentration change vectors weighed by an also diagonal matrix ***R***.

The feedback is controlled according to the equations below (the derivation of Equations (9)–(12) are written in Appendix A). $K_{k}$ can be considered as a feedback gain matrix at time *k*, which controls concentrations according to the magnitude of ($s_{k}-s_{target}$). $K_{k}$ is adjusted at each time.

(9) $$u_{k}=-K_{k}\left(s_{k}-s_{target}\right),$$

$$\mathrm{Initial}\;\mathrm{matrix}:\;M_{p}=Q,$$

$$\mathrm{from}\;i=\left(p-1\right)\;\mathrm{to}\;i=1$$

(10) $$K_{i}={\left(G^{T}M_{i+1}G+R\right)}^{-1}G^{T}M_{i+1}F,$$

(11) $$P_{i}=F^{T}M_{i+1}\left(F-GK_{i}\right),$$

(12) $$M_{i}=Q+P.$$

### 2.7. Preprocessing for Concentration-Change Track with Feedback

Principal component analysis (PCA) was utilized for dimensional reduction to obtain the $s_{k}$ as an alternative to sensor response vector. The same number of QCM sensors as the number of components of the gas mixture was used previously [23]. Components between the first component and the third one were utilized for $s_{k}$ in this work. There is much information in data of FAIMS (the number of DFs can be selected from 1 to 51, and the number of CVs can be selected from 1 to 512). Four DF (51.1%, 55.3%, 59.5%, and 63.7%) values were utilized in this study for PCA analysis. The data obtained from those values were realigned to one line. Thus, the dimension of the data for PCA was 2048. One hundred twenty-five data were used for making models. Measurement concentrations were chosen from a combination of $x_{1}$, $x_{2}$, and $x_{3}$ (see Table 4). The three-target concentrations were selected (see Table 5). The weight matrices such as Qs were found using an exhaustive search.

## 3. Results

**Figure 7:**
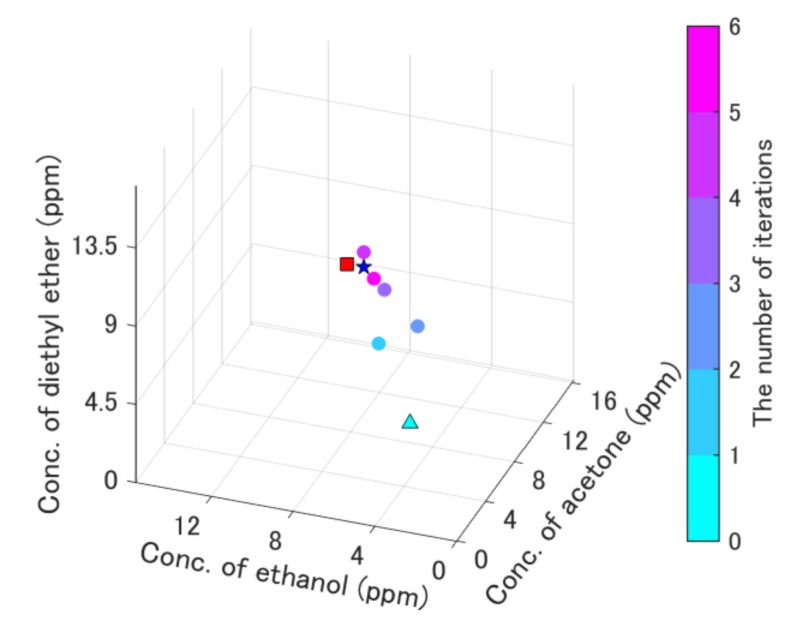
Results of the on-line quantification of the ternary gas mixture after the concentration-level quantization. The light blue triangle is the initial point, the red square is the target point, and the blue star is the point with the smallest error value, which was the solution of the quantification. The orange points were used to calculate the gradient. The history of the update points was plotted and colored with the number of iterations. Refer to data in detail in the supplemental files (Figures S1 and S2).

First, the performance of the on-line quantification system was confirmed. The results of the quantification to the ternary gas mixture after the concentration-level quantization are shown in Figure 7. The measurement history of the update points and measured points are shown in Figure 7. The result indicates that the update point moved directly to the target point placed in the center, and finally arrived at the point near the target one. The quantification of the ternary mixture was successful, as is shown in Figure 7. The total time for quantification was 635 s.

**Figure 8:**
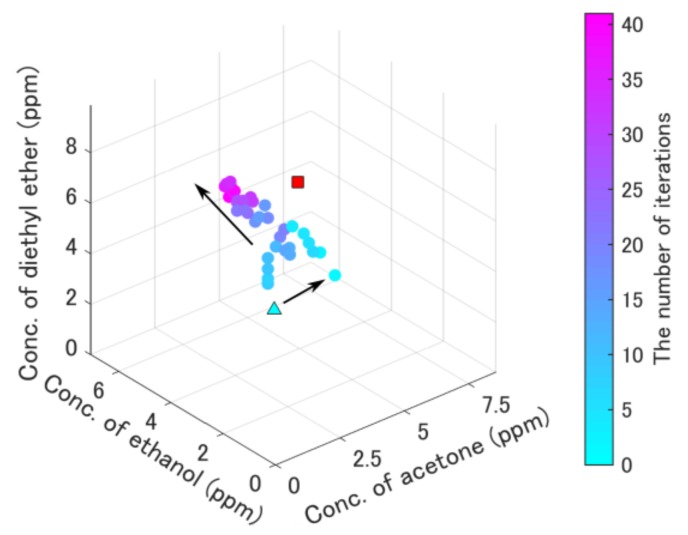
The result of on-line quantification without the quantization method. The light blue triangle is the initial point, and the red square is the target point. The arrows in this figure were different from the true direction of the gradient owing to the noise caused by the flowrate fluctuation.

Figure 8 shows a failure example of quantification without quantization. The arrows in this figure were different from the true direction of the gradient owing to the noise caused by the flowrate fluctuation. The quantification result is different from the target one, because the gradient vector was inappropriate without quantization.

**Figure 9:**
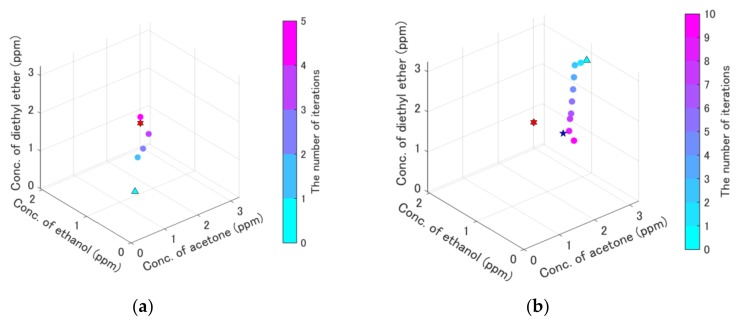
Results of on-line quantification for ternary gas mixture with sub-ppb-level concentrations. The light blue triangle is the initial point, and the red hexagram is the target point, which is the same as the point with the smallest error in (**a**). The point with the smallest error in (**b**) is the blue star. The history of the update points was plotted colored with the number of iterations. Refer to data in detail in the supplemental files (Figures S3–S6). (**a**) Success in quantification. (**b**) Failure in quantification.

A ternary gas mixture with sub-ppb-level concentrations was also examined. Figure 9 shows the results of the quantification of the ternary gas mixture with the sub-ppb-level concentrations. Figure 9a shows the result of successful quantification, whereas Figure 9b shows the result of a failure in quantification due to a local minimum. The smallest error value $E_{gm}$ in Figure 9a was 3.8, whereas that in Figure 9b was 7.4. Specific information about Figure 7, Figure 8 and Figure 9 is summarized in Table 6.

**Figure 10:**
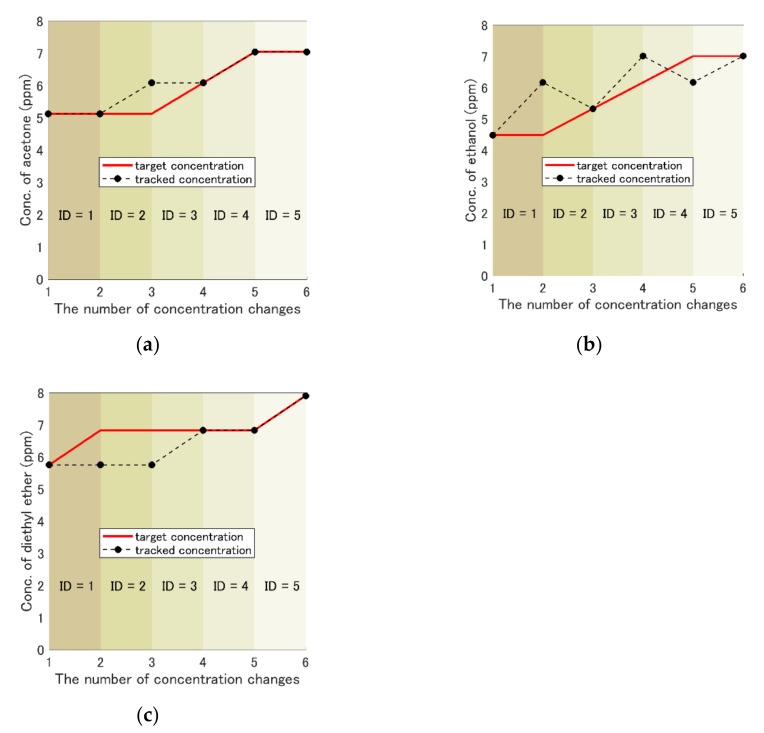
Results of the concentration-change track with the gradient descent method. Each component of the ternary gas mixture was individually plotted for easy visualization. The black dots the history of the concentration changes, and the bold red line is the concentration of the target. Initial concentrations were acetone (5.1 ppm), ethanol (4.5 ppm), and diethyl ether (5.8 ppm). The temporal concentration changes were selected randomly from one quantization point (1.0 ppm of acetone, 0.8 ppm of ethanol, and 1.1 ppm of diethyl ether) or 0 ppm for each component at the same time. ID indicates each quantification, and the required times were written in Table 7. (**a**) Acetone. (**b**) Ethanol. (**c**) Diethyl ether.

Next, we aimed to track the temporal change of each component’s concentration using the gradient descent method, as is shown in Figure 10a–c. The initial concentrations were acetone (5.1 ppm), ethanol (4.5 ppm), and diethyl ether (5.8 ppm). This result indicates that the quantified concentration of each gas followed roughly the concentration changes in spite of certain errors. The time required to complete the quantification is shown in Table 7.

**Figure 11:**
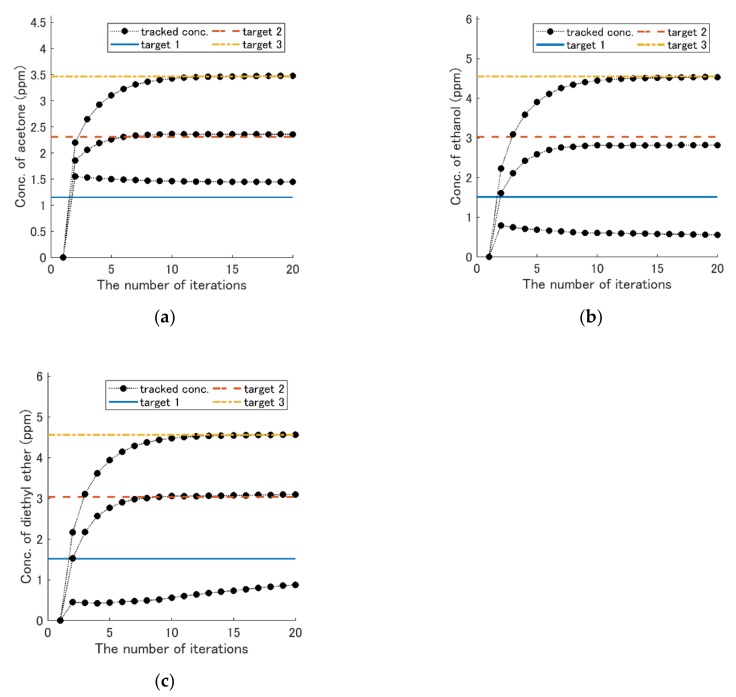
Results of the concentration-change track with the feedback using weight matrix $Q_{1}$. The solid blue line is the concentration of target 1, the broken red line is the concentration of target 2, and the dash–dot orange line is the concentration of target 3 (Table 5). The black dots are the history of the concentration changes. (**a**) Acetone. (**b**) Ethanol. (**c**) Diethyl ether.

**Figure 12:**
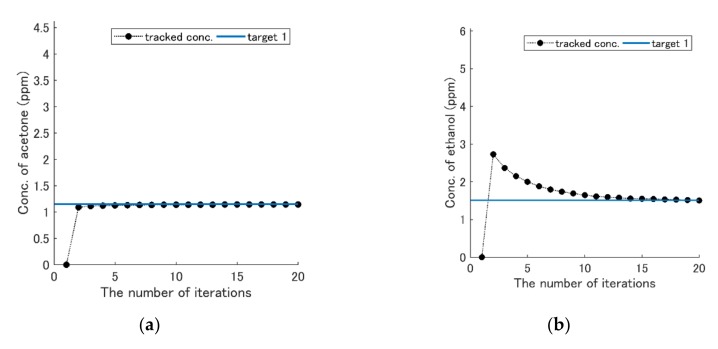

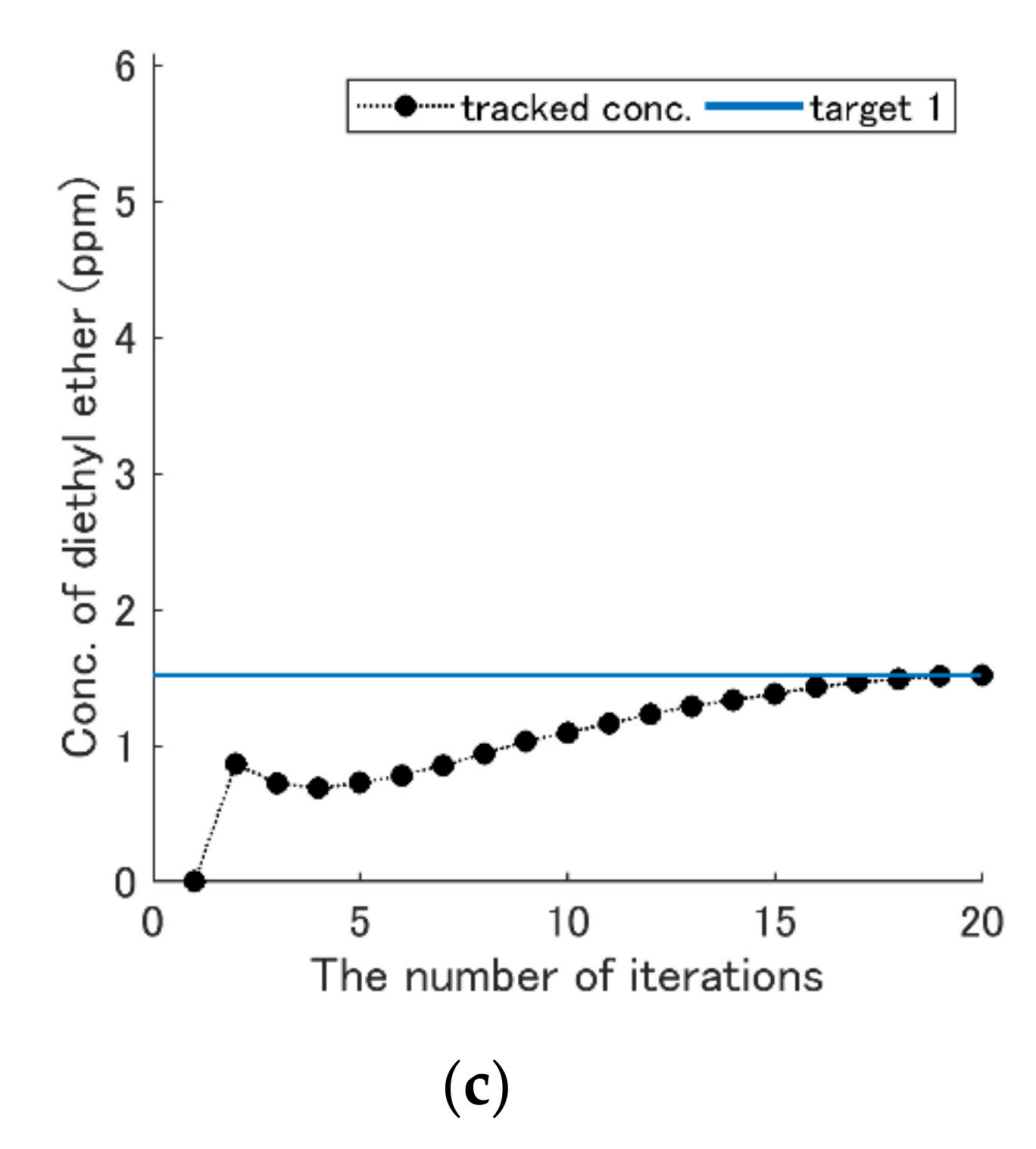
Results of the concentration-change track with the feedback using weight matrix $Q_{2}$. The solid blue line is the concentration of target 1 (Table 5), and the black dots are the history of the concentration changes. (**a**) Acetone. (**b**) Ethanol. (**c**) Diethyl ether.

**Figure 13:**
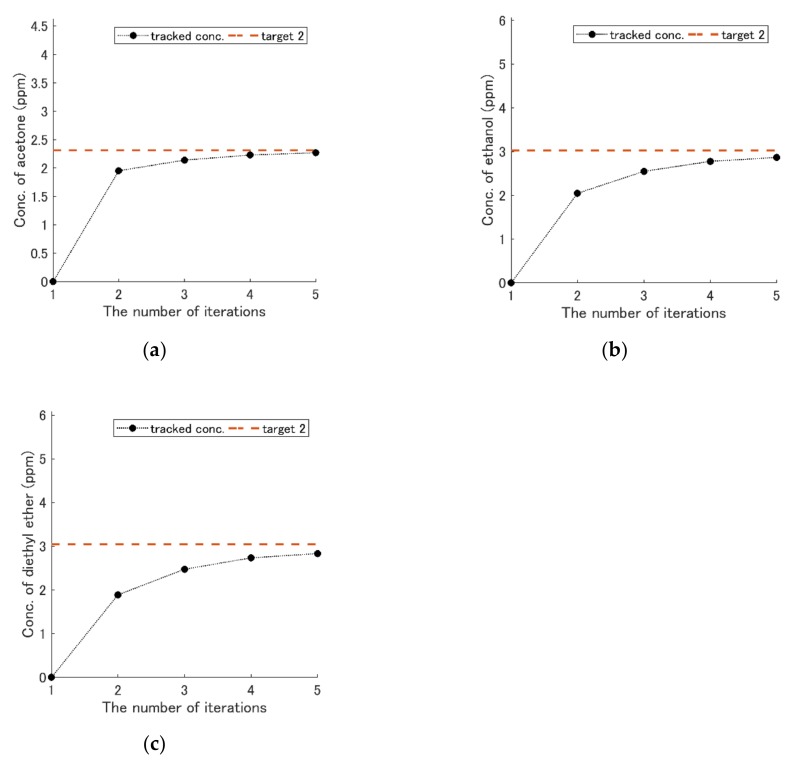
Results of the concentration-change track with the feedback using weight matrix $Q_{3}$. The number of iterations was changed to five times from 20 times. The broken red line is the concentration of target 2, and the black dots are the history of the concentration changes in each figure. (**a**) Acetone. (**b**) Ethanol. (**c**) Diethyl ether.

Then, the feedback method was needed to reduce the quantification time. The simulation results of the concentration-change track with the feedback method are shown in Figure 11, Figure 12 and Figure 13. The concentration of each component was kept constant during the experiment in this study. The three types of the first term weight matrix Qs were selected:

(13) $$Q_{1}=\left(\begin{array}{ccc} 1 & 0 & 0\\ 0 & 1.1 & 0\\ 0 & 0 & 1.7 \end{array}\right),\;Q_{2}=\left(\begin{array}{ccc} 7.5 & 0 & 0\\ 0 & 13 & 0\\ 0 & 0 & 4.7 \end{array}\right),Q_{3}=\left(\begin{array}{ccc} 1.8 & 0 & 0\\ 0 & 1.4 & 0\\ 0 & 0 & 1.7 \end{array}\right).$$

The second term of weight matrix R was set to 50I, where I is an identity matrix. The accuracy of the concentration-change track with $Q_{1}$ was worse than others when the target concentration was low (see Figure 11). However, using $Q_{2}$, the accuracy was improved (see Figure 12). Figure 13 shows the result with $Q_{3}$. The number of iterations was reduced to five times.

## 4. Discussion

When we measure the all possible combinations of component concentrations, the measurement time increases exponentially. This is one of the reasons for developing the on-line system. The ternary mixture quantification needs 2–3 h in this situation. The on-line measurement reduces the time drastically, since its exploratory area in concentration space is quite limited. Thus, the measurement time can be reduced from 3 h to 10 min. In order to further reduce the quantification time, the cleaning process should be skipped. Moreover, the alternate measurement of the target gas and blended gas repeatedly is useful to eliminate the background fluctuation.

The reason for successful on-line quantification (Figure 7 and Figure 9a) was due to the appropriate quantization. If the concentrations were not quantized, the update points moved in the wrong direction due to the error (Figure 8). On the contrary, the update point (Figure 7) went straight to the target point. The result in Figure 8 indicates that estimated gradients were inappropriate because the update point seemed to be irregularly moved, although it moved toward the inappropriate direction. Thus, the quantization of an appropriate concentration level is necessary.

The reason that the update point in Figure 9b did not reach the target point is due to a local minimum because the smallest error value $E_{lm}$ in Figure 9b (= 7.4) was still high compared with smallest error value $E_{gm}$ (= 3.8) in Figure 9a, which is almost the global minimum. The difference between $E_{lm}\;$ and $E_{gm}$ can be utilized to detect the local minimum. Even if the initial point in Figure 9b is used, the target point concentrations (the red hexagram in Figure 9a) can be obtained when the local minimum detection triggers the jump to the area outside the local minimum.

We also confirmed the capability to track the temporal change of each component concentration in the mixture, as is shown in Figure 10. However, the large concentration changes per each step were not allowed, because it took a long time to find the solution when those changes were large. Thus, the track of temporal change of each component concentration with the feedback method was considered in order to track faster changes of the concentrations without any limitations.

When the target concentrations were larger than certain levels, the simulation with the feedback method was successful (Figure 11), because the tracked concentrations became approximately the same concentrations as the target ones. Although the accuracy deteriorated when the target concentrations became lower, it was improved using $Q_{2}$. The optimal Q depends on the target concentrations. The advantage of the feedback method over the gradient descent method is to reduce the number of measurement points. The number of iterations was reduced from 20 to 5 when $Q_{3}$ was used (see Figure 13). The time may decrease down to two minutes and 30 seconds with actual measurement, because around 20 iterations in Figure 7 took 10 minutes. Although the linear model was used here, a nonlinear model might further enhance its performance. Recently, Sun et al. utilized the machine learning method for clinical wound detection, and it achieved high-detection rates [24]. Therefore, if machine learning methods can be combined with this method for feature extraction, it will improve the performance.

## 5. Conclusions

Recently, FAIMS is mainly used for the integration of MS [25,26,27] or solving classification problems for medical disease analysis [28]. Our method to quantify mixtures is a new approach to the application of FAIMS. Our quantification method needs only FAIMS without any other expensive measurement equipment. This paper reports the on-line quantification for ternary gas mixtures using FAIMS and the simulation of odor tracking using FAIMS for the feasibility of real-time quantification using FAIMS. The on-line quantification using FAIMS was successful. Moreover, on-line quantification for sub-ppb-level concentrations was demonstrated. The feasibility of tracking of the temporal concentration changes with the feedback method was exhibited. In the future, this system can integrate olfactory display, and it can be used for an odor replicate system. FAIMS can also be utilized for odor replication in virtual reality. Moreover, this approach can be applied to an odor-tracking robot. Kostyukevich et al. reported odor tracking using a drone with FAIMS [29]. We will make the mixture quantification with FAIMS more sophisticated in future studies.

## Figures and Tables

**Table 1:** The explanation of the protocol of quantification simulation using all possible combinations of component concentrations. FAIMS: field asymmetric ion mobility spectrometry.

Procedure	Procedure Explanation
1	Check the blank with sampling bag air using FAIMS.
2	Put the gas into a sampling bag. Measure gas concentration in sampling using a photoionization detector.
3	Perform FAIMS measurement at every lattice points (It takes 2–3 h).
4	Calculate index *E* at every lattice point.
5	Obtain error hypersurface using linear interpolation.
6	Select initial concentrations as an update point and target concentrations. If the target point is not on the lattice, it should be measured.
7	Error surface is obtained from index *E* using linear interpolation.
8	Calculate a gradient, and move the update point to the direction repeatedly until the number of iterations reaches an allowable number of times.

**Table 2:** The explanation of the protocol of on-line quantification using FAIMS.

Procedure	Procedure Explanation
1	Check the blank with sampling bag air using FAIMS.
2	Put the gas into a sampling bag. Measure gas concentration in sampling using a photoionization detector.
3	Perform FAIMS measurement of a target gas mixture.
4	Select initial-point concentrations as an update point.
5	Select points close to the update point to calculate the gradient.
6	Perform a FAIMS measurement of selected new points.
7	Calculate index *E* at the update point and selected points according to Equation (2).
8	Calculate a gradient, and move the update point to the direction little by little.
9	When stagnation is detected, go to 10. Otherwise, go to 5.
10	When the minimum of *E* in the historical record is below a threshold, finish quantification. Otherwise, go to 4.

**Table 3:** Explanation of data measurement protocol. DF: dispersion field, MFC: mass flow controller.

Procedure	Procedure Explanation	Required Time (s)
1	Clean FAIMS device	10
2	Change flowrate of MFC	Immediately
3	Wait for mixing gases	depending on flowrates
4	Data measurement with FAIMS	3–90 (depending on the number of DF)

**Table 4:** Data explanation for tracking concentration change with feedback.

Name	Concentration Candidates (ppm)
$x_{1}$(acetone)	0, 1.2, 2.3, 3.5, 4.6
$x_{2}$(ethanol)	0, 1.5, 3.0, 4.5, 6.1
$x_{3}$(diethyl ether)	0, 1.5, 3.0, 4.6, 6.1

**Table 5:** The three types of target concentrations for tracking concentrations change with feedback.

ID of Target Concentrations	Acetone (ppm)	Ethanol (ppm)	Diethyl Ether (ppm)
target 1	1.2	1.5	1.5
target 2	2.3	3.0	3.0
target 3	3.5	4.5	4.6

**Table 6:** Specific information in Figure 7, Figure 8 and Figure 9.

	Name	Figure 7	Figure 8	Figure 9a	Figure 9b
Target concentrations (red square and red hexagram)	Acetone (ppm)	8.7	5.4	1.8	1.8
	Ethanol (ppm)	8.4	4.4	1.1	1.1
	Diethyl ether (ppm)	9.1	6.2	1.7	1.7
Initial-point concentrations (light blue triangle)	Acetone (ppm)	3.7	3.0	0.8	3.3
	Ethanol (ppm)	3.6	3.0	0.5	1.1
	Diethyl ether (ppm)	3.9	3.0	0.7	2.7
Smallest error concentrations (blue star and red hexagram)	Acetone (ppm)	8.7	-	1.8	1.8
	Ethanol (ppm)	7.6		1.1	0.5
	Diethyl ether (ppm)	9.1		1.7	1.9
Resolution of concentrations in quantization	Acetone (ppm)	0.83	-	0.17	0.17
	Ethanol (ppm)	0.81		0.1	0.1
	Diethyl ether (ppm)	0.88		0.17	0.17
Maximum concentration of measurement range	Acetone (ppm)	16.2	8.6	3.3	3.3
	Ethanol (ppm)	15.7	7.1	2.0	2.0
	Diethyl ether (ppm)	17.0	9.9	3.2	3.2
Minimum concentration of measurement range	Acetone (ppm)	2.0	2.2	0.42	0.42
	Ethanol (ppm)	2.0	1.8	0.27	0.27
	Diethyl ether (ppm)	2.1	2.5	0.42	0.42

**Table 7:** The result of the required time. ID indicates each quantification in Figure 10.

ID	Time (s)
1	310
2	93
3	154
4	62
5	92

## Supplementary Materials

The following are available online at https://www.mdpi.com/1424-8220/19/24/5442/s1, Figure S1: 2D-view of Figure 7, Figure S2: 2D-view of Figure 7 with all of collected points, Figure S3: 2D-view of Figure 9a, Figure S4: 2D-view of Figure 9a with all of collected points, Figure S5: 2D-view of Figure 9b, Figure S6: 2D-view of Figure 9b with all of collected points.

## Appendix A

The derivations of Equations (9)–(12) are shown in below [23]. The difference between $s_{k+1}$ and $s_{target}$ is written in the form:

(A1) $$s_{k+1}-s_{target}=F\left(s_{k}-s_{target}\right)+Gu_{k},$$

where $s_{k}$ is the sensor response vector to the blended at time *k*T, and $s_{target}$ is the sensor response vector to the target gas mixture. The index value to be minimized is defined as

(A2) $$J_{p-1}={\left(s_{p}-s_{target}\right)}^{T}Q\left(s_{p}-s_{target}\right)+u_{p-1}^{T}Ru_{p-1}.$$

The index value $J_{p-1}$ at time (*p*−1)T is expressed using Equations (A1) and (A2):

(A3) $$J_{p-1}={\left\{F\left(s_{p-1}-s_{target}\right)+Gu_{p-1}\right\}}^{T}Q\left\{F\left(s_{p-1}-s_{target}\right)+Gu_{p-1}\right\}+u_{p-1}^{T}Ru_{p-1}.$$

When $J_{p-1}$ is differentiated with respect to $u_{p-1}$, the following equation is obtained:

(A4) $$\frac{\partial J_{p-1}}{\partial u_{p-1}}=2G^{T}Q\left\{F\left(s_{p-1}-s_{target}\right)+Gu_{p-1}\right\}+2Ru_{p-1}.$$

$u_{p-1}$ is obtained as the solution to minimize the $J_{p-1}$ if the Equation (A4) is set to zero:

(A5) $$u_{p-1}=-{\left(G^{T}QG+R\right)}^{-1}G^{T}QF\left(s_{p-1}-s_{target}\right).$$

When the feedback gain matrix $K_{p-1}$ at the time (*p*−1)T is defined by

(A6) $$K_{p-1}={\left(G^{T}QG+R\right)}^{-1}G^{T}QF,$$

Equation (A5) is transformed to next equation:

(A7) $$u_{p-1}=-K_{p-1}\left(s_{p-1}-s_{target}\right).$$

If $u_{p-1}$ in Equation (A7) is substituted into Equation (A5) from Equation (A3), $J_{p-1}$ is expressed the following equation:

(A8) $$\begin{array}{ll} J_{p-1} & ={\left\{F\left(s_{p-1}-s_{target}\right)-GK_{p-1}\left(s_{p-1}-s_{target}\right)\right\}}^{T}Q\{F\left(s_{p-1}-s_{target}\right)\\ & -GK_{p-1}\left(s_{p-1}-s_{target}\right)\}+{\left(s_{p-1}-s_{target}\right)}^{T}K_{p-1}^{T}RK_{p-1}\left(s_{p-1}-s_{target}\right)\\ & ={\left(s_{p-1}-s_{target}\right)}^{T}{\left\{F-GK_{p-1}\right)}^{T}Q\left(F-GK_{p-1}\right)+K_{p-1}^{T}RK_{p-1}\}\left(s_{p-1}-s_{target}\right)\\ & ={\left(s_{p-1}-s_{target}\right)}^{T}\left\{F^{T}Q\left(F-GK_{p-1}\right)-K_{p-1}^{T}G^{T}QF+K_{p-1}^{T}\left(G^{T}QG+R\right)K_{p-1}\right\}\left(s_{p-1}-s_{target}\right). \end{array}$$

Equation (A8) can be transformed using Equation (A6):

(A9) $$\begin{array}{ll} J_{p-1} & ={\left(s_{p-1}-s_{target}\right)}^{T}\left\{F^{T}Q\left(F-GK_{p-1}\right)-K_{p-1}^{T}G^{T}QF+K_{p-1}^{T}G^{T}QF\right\}\left(s_{p-1}-s_{target}\right)\\ & ={\left(s_{p-1}-s_{target}\right)}^{T}F^{T}Q\left(F-GK_{p-1}\right)\left(s_{p-1}-s_{target}\right) \end{array}$$

If $P_{p-1}$ is defined as

(A10) $$P_{p-1}=F^{T}Q\left(F-GK_{p-1}\right),$$

then Equation (A11) is obtained:

(A11) $$J_{p-1}={\left(s_{p-1}-s_{target}\right)}^{T}P_{p-1}\left(s_{p-1}-s_{target}\right).$$

Let us consider the index value $J_{p-2}$ at time (*p*−2)T. The equation is given by

(A12) $$J_{p-2}={\left(s_{p-1}-s_{target}\right)}^{T}Q\left(s_{p-1}-s_{target}\right)+u_{p-2}^{T}Ru_{p-2}+J_{p-1}$$

using Equation (A8). If Equation (A12) is differentiated with respect to $u_{p-2}$, the following equation is obtained:

(A13) $$\frac{\partial J_{p-2}}{\partial u_{p-2}}=2G^{T}Q\left\{F\left(s_{p-2}-s_{target}\right)+Gu_{p-2}\right\}+2Ru_{p-2}+\frac{\partial J_{p-1}}{\partial u_{p-2}}.$$

The differentiation of $J_{p-1}$ with respect to $u_{p-2}$ is given by

(A14) $$\begin{array}{ll} \frac{\partial J_{p-1}}{\partial u_{p-2}} & =\frac{\partial }{\partial u_{p-2}}\left[{\left\{F\left(s_{p-2}-s_{target}\right)+Gu_{p-2}\right\}}^{T}P_{p-1}\left\{F\left(s_{p-2}-s_{target}\right)+Gu_{p-2}\right\}\right]\\ & =2G^{T}P_{p-1}\left\{F\left(s_{p-2}-s_{target}\right)+Gu_{p-2}\right\} \end{array}$$

using Equation (A11). Therefore, the following equation is obtained using Equation (A14) if Equation (A13) set to zero:

(A15) $$\begin{array}{ll} u_{p-2} & =-{\left\{G^{T}\left(Q+P_{p-1}\right)G+R\right\}}^{-1}G^{T}\left(Q+P_{p-1}\right)F\left(s_{p-2}-s_{target}\right)\\ & =-{\left(G^{T}M_{p-1}G+R\right)}^{-1}G^{T}M_{p-1}F\left(s_{p-2}-s_{target}\right)\\ & =-K_{p-2}\left(s_{p-2}-s_{target}\right), \end{array}$$

where

(A16) $$M_{p-1}=Q+P_{p-1}$$

(A17) $$K_{p-2}={\left(G^{T}M_{p-1}G+R\right)}^{-1}G^{T}M_{p-1}F.$$

$J_{p-2}$ can be obtained using Equation (A16):

(A18) $$\begin{array}{ll} J_{p-2} & ={\left(s_{p-1}-s_{target}\right)}^{T}Q\left(s_{p-1}-s_{target}\right)+u_{p-2}^{T}Ru_{p-2}\\ & +{\left(s_{p-1}-s_{target}\right)}^{T}P_{p-1}\left(s_{p-1}-s_{target}\right)\\ & ={\left(s_{p-1}-s_{target}\right)}^{T}\left(Q+P_{p-1}\right)\left(s_{p-1}-s_{target}\right)+u_{p-2}^{T}Ru_{p-2}\\ & ={\left(s_{p-1}-s_{target}\right)}^{T}M_{p-1}\left(s_{p-1}-s_{target}\right)+u_{p-2}^{T}Ru_{p-2.} \end{array}$$

Comparing Equation (A18) with (A2), $J_{p-2}$ can be transformed in the same manner as the calculation for the $J_{p-1}$ (Equations (A8)–(A11)):

(A19) $$J_{p-2}={\left(s_{p-2}-s_{target}\right)}^{T}P_{p-2}\left(s_{p-2}-s_{target}\right),$$

where

(A20) $$P_{p-2}=F^{T}M_{p-1}\left(F-GK_{p-2}\right).$$

The calculation is repeated from the time (*p-2*) T to the time 0. Therefore, Equations (9)–(12) are obtained.
