# Supplementary material for: On-Line Mixture Quantification to Track Temporal Change of Composition Using FAIMS
Source: Sensors (Basel). 2019 Dec 10;19(24):5442. doi: 10.3390/s19245442 (PMC6960543; doi:10.3390/s19245442)
Supplement: Supplementary file 1 [file sensors-19-05442-s001.pdf]

sensors

MDPI

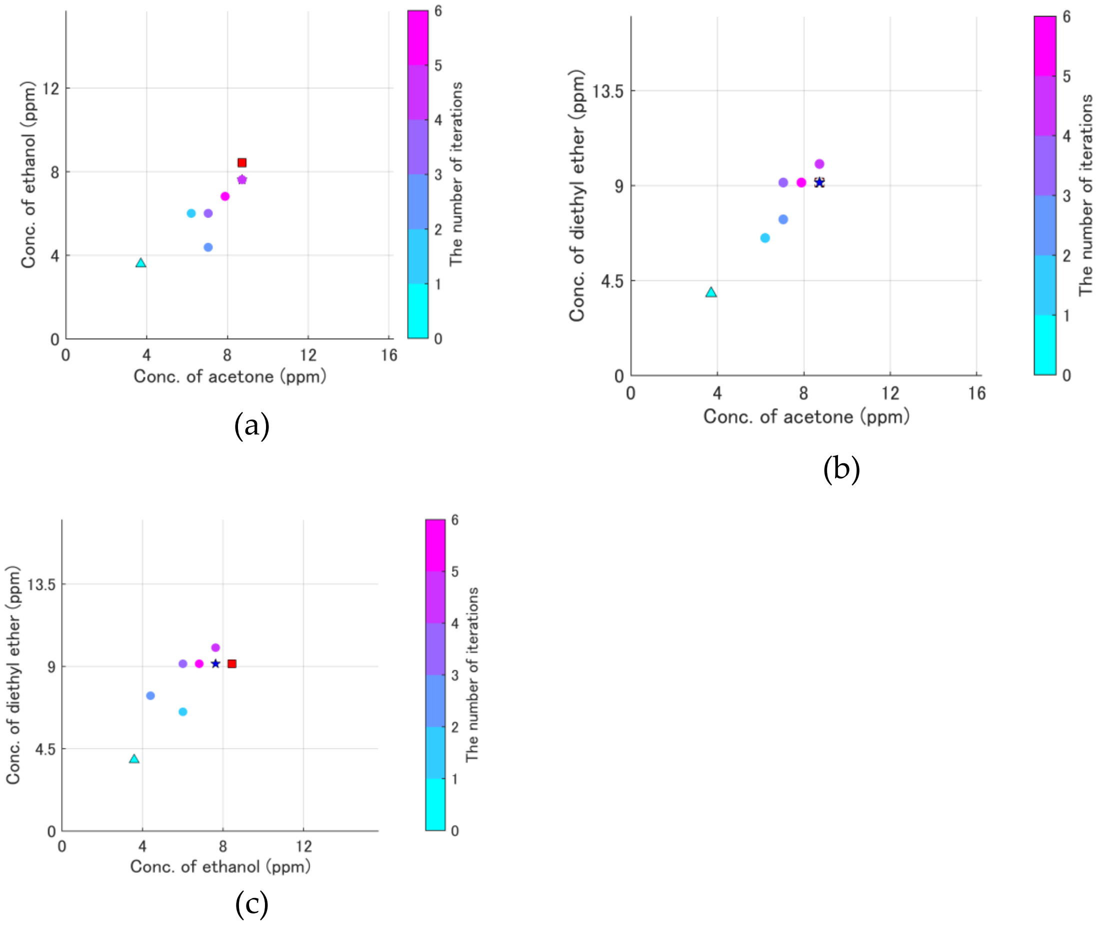

Figure S1. 2D-view of results of the on-line quantification of the ternary gas mixture. Results of the on-line quantification of the ternary gas mixture after the concentration-level quantization. The light blue triangle is the initial point, the red square is the target point, and the blue star is the point with the smallest error value, which was the solution of the quantification. The history of the update points was plotted colored with the number of iterations. (a) Acetone-ethanol plane. (b) Acetone-diethyl-ether plane. (c) Ethanol-diethyl-ether plane.

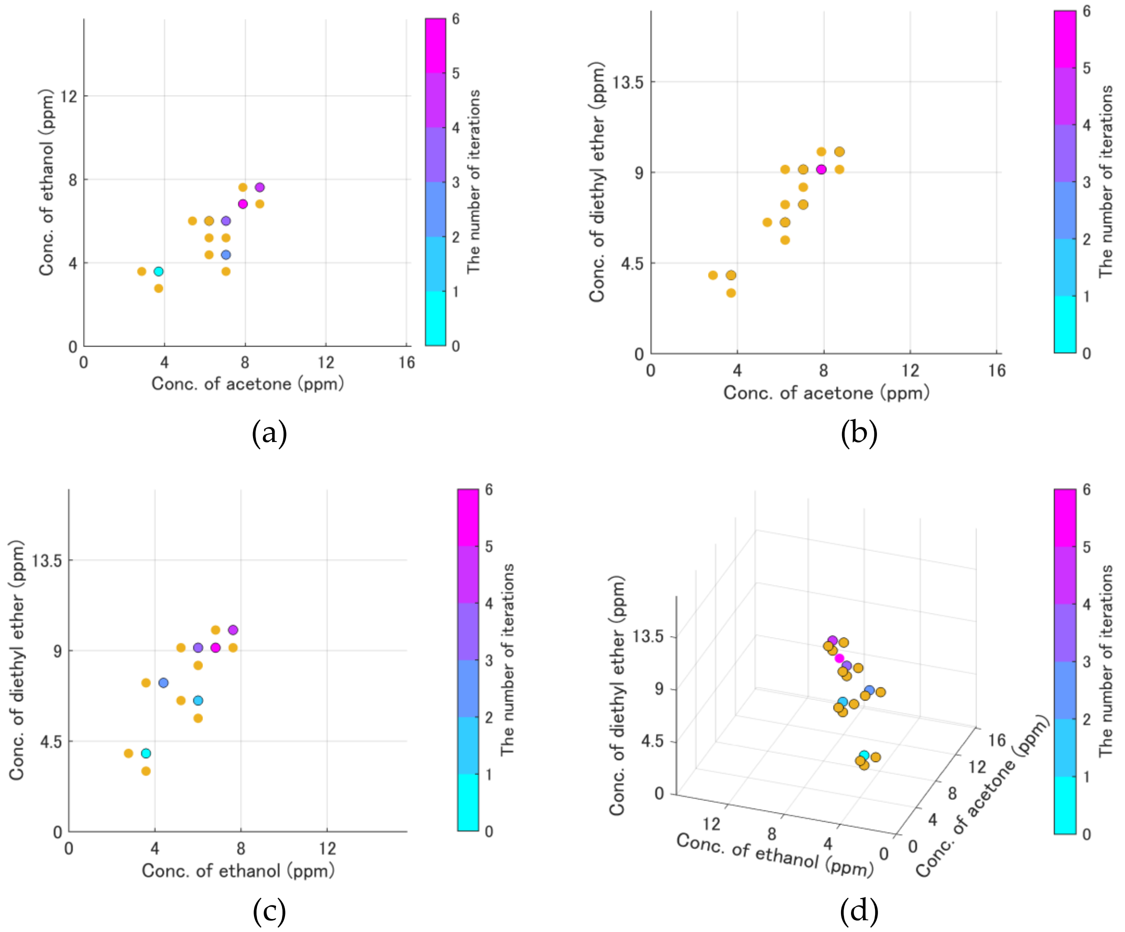

Figure S2. 2D-view of results of the on-line quantification of the ternary gas mixture. All of the measured points are shown in the figures. The orange points were used to calculate the gradient (collected points). (a) Acetone-ethanol plane. (b) Acetone-diethyl-ether plane. (c) Ethanol-diethyl-ether plane (d) 3D-View.

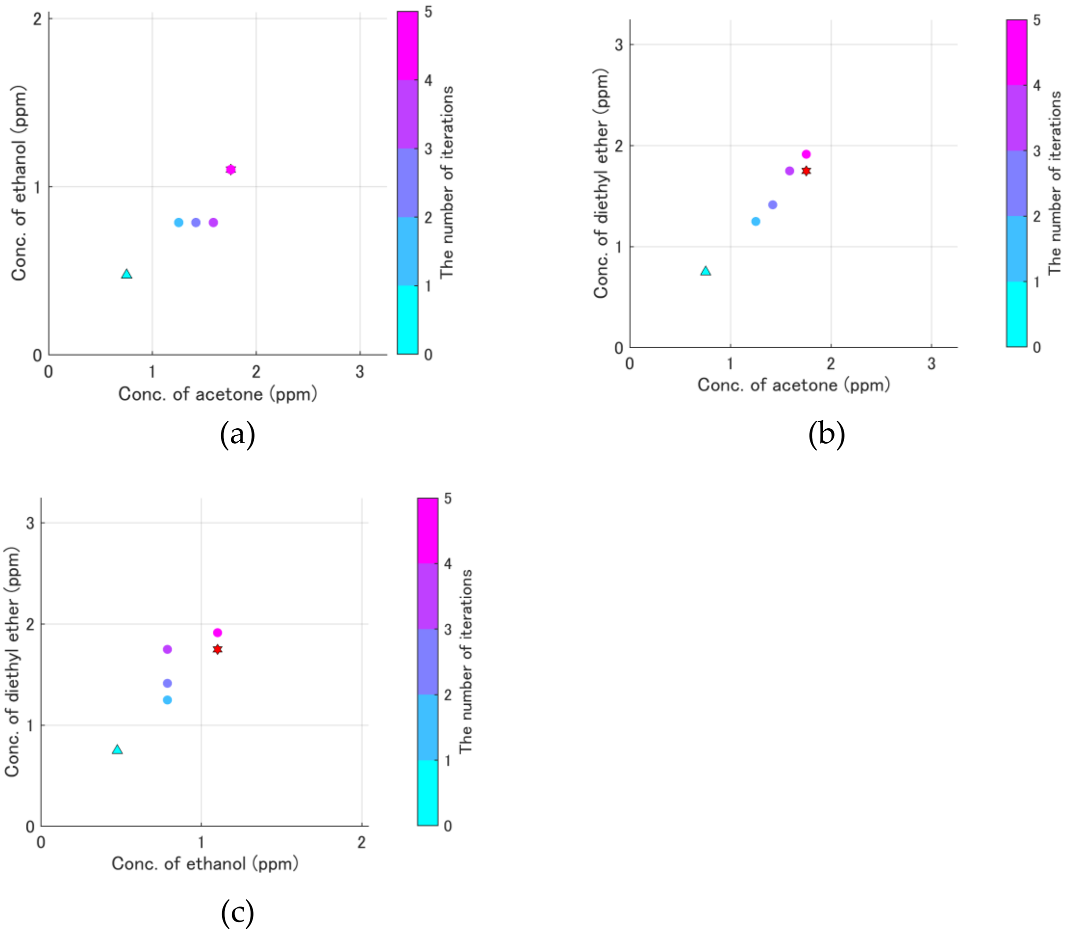

Figure S3. 2D-view of results of on-line quantification for ternary gas mixture with sub-ppb-level concentrations. The light blue triangle is the initial point, and the red hexagram is the target point the same as the point with the smallest error. The history of the update points was plotted colored with the number of iterations. (a) Acetone-ethanol plane. (b) Acetone-diethyl-ether plane. (c) Ethanol-diethyl-ether plane.

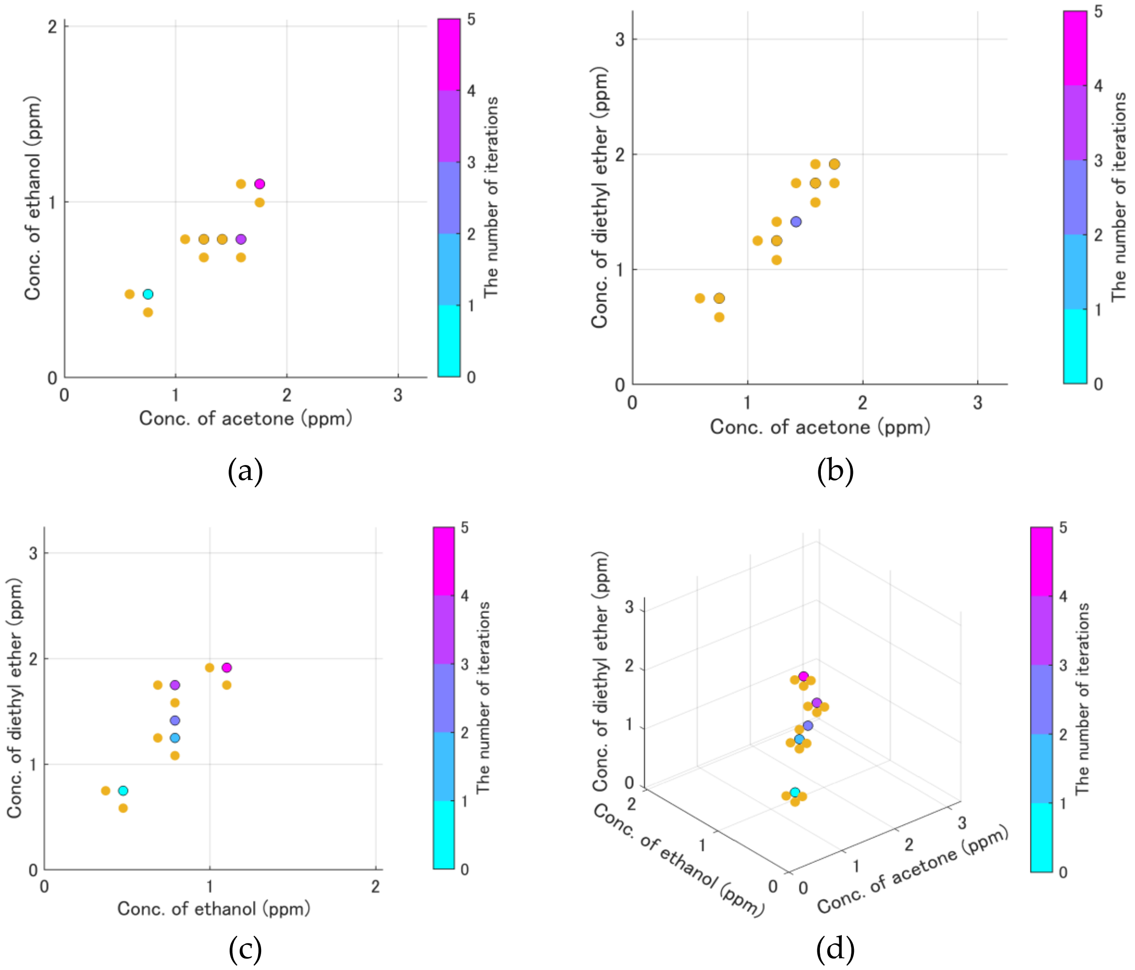

Figure S4. 2D-view of results of on-line quantification for ternary gas mixture with sub-ppb-level concentrations. All of the measured points were shown in the figures. The orange points were used to calculate the gradient (collected points). (a) Acetone-ethanol plane. (b) Acetone-diethyl-ether plane. (c) Ethanol-diethyl-ether plane. (d) 3D-view.

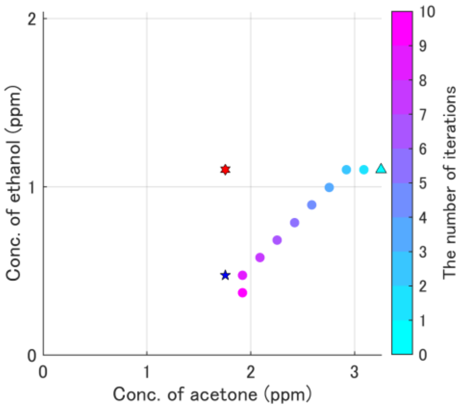

(a)

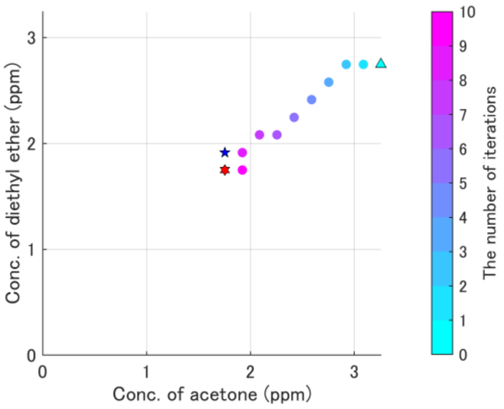

(b)

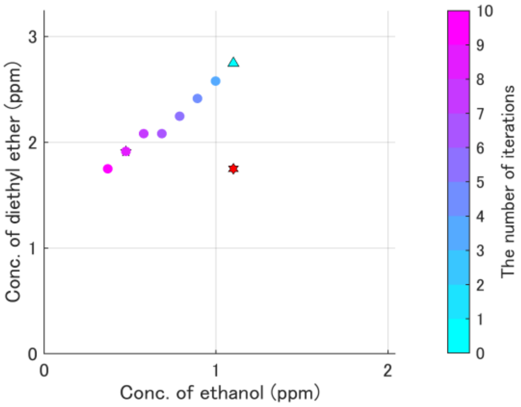

(c)

Figure S5. 2D-view of results of on-line quantification for ternary gas mixture with sub-ppb-level concentrations. The light blue triangle is the initial point, and the red hexagram is the target point. The point with the smallest error is the blue star. The history of the update points was plotted colored with the number of iterations. (a) Acetone-ethanol plane. (b) Acetone-diethyl-ether plane. (c) Ethanol-diethyl-ether plane.

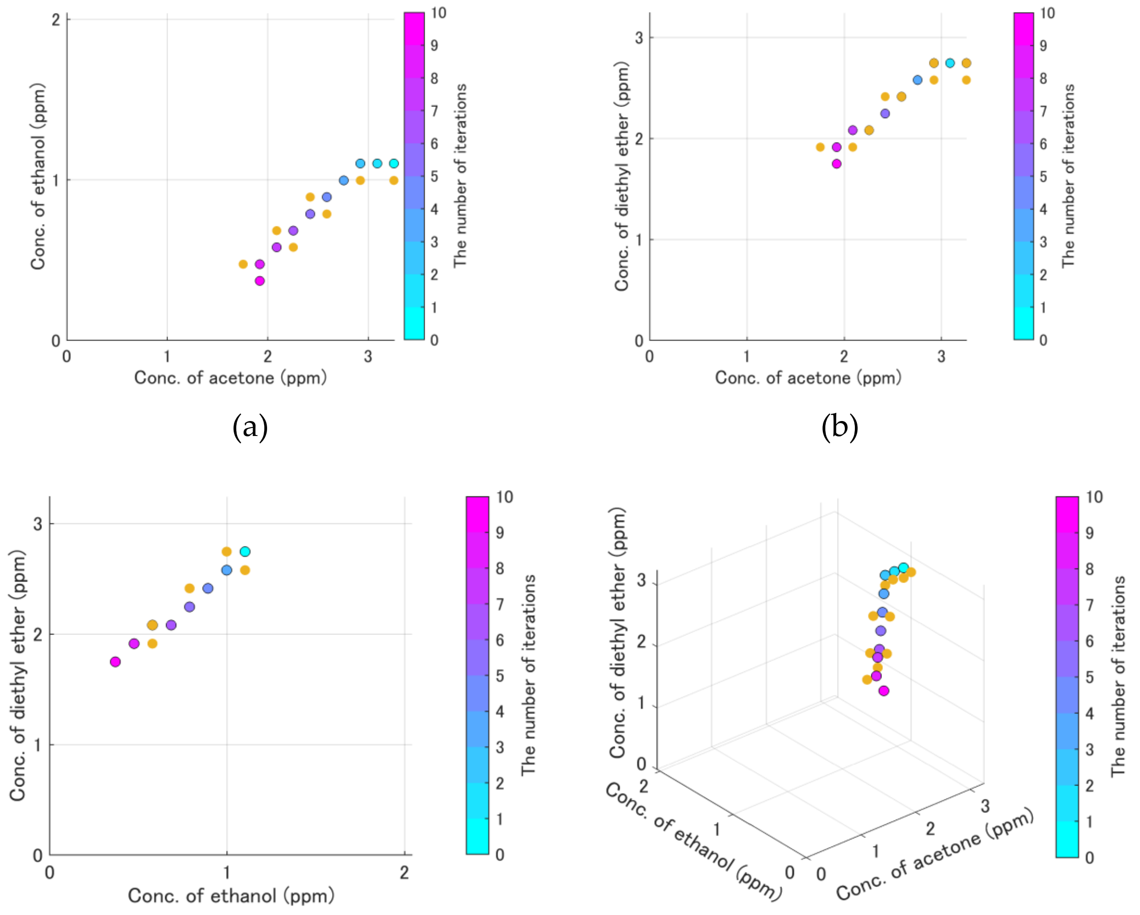

(a) (b)

(c) (d)

Figure S6. 2D-view of results of on-line quantification for ternary gas mixture with sub-ppb-level concentrations. All of the measured points are shown in the figures. The orange points were used to calculate the gradient (collected points). (a) Acetone-ethanol plane. (b) Acetone-diethyl-ether plane. (c) Ethanol-diethyl-ether plane. (d) 3D-view.
